# Butyrylcholinesterase as a potential biomarker of metabolic imbalances in Afro-descendant populations

**DOI:** 10.1038/s41366-026-02095-2

**Published:** 2026-05-13

**Authors:** Denise Raquel de Moura Bones, Marcia Machado Ramos, Natali Fátima Veigand, Iriel Araceli Joerin-Luque, Natalie Mary Sukow, Sarah Elisabeth Cupertino, Priscila Ianzen dos Santos, Hellen Karoline de Oliveira Nunes, Aymee Fernanda Gros, Victor Dobis Barros, Joana Gehlen Tessaro, Luana Leonardo Garcia, Isabela Dall Oglio Bucco, Letícia Boslooper Gonçalves, Ana Cecília Guimarães Alves, Thalia Roberta de Moraes, Selma Árabe Andrietta, Thomas Farias de Cristo, Vanessa dos Santos Ribeiro Zanette, Adriana Inês de Paula, Aline Borsato Hauser, Claudemira Vieira Gusmão Lopes, Luciane Viater Turek, Lupe Furtado Alle, Ricardo Lehtonen Rodrigues Souza, Marcia Holsbach Beltrame

**Affiliations:** 1 Programa de Pós-graduação em Genética, Departamento de Genética, Universidade Federal do Paraná (UFPR), Curitiba, Brazil; 2 Laboratório de Genética Molecular Humana, Departamento de Genética, Universidade Federal do Paraná (UFPR), Curitiba, Brazil; 3 Laboratório de Polimorfismos e Ligação, Departamento de Genética, Universidade Federal do Paraná (UFPR), Curitiba, Brazil; 4 Programa de Pós-graduação em Medicina Interna, Hospital de Clínicas (HC), Universidade Federal do Paraná (UFPR), Curitiba, Brazil; 5 Departamento de Educação Física, Universidade Federal do Paraná (UFPR), Curitiba, Brazil; 6 Laboratório Escola de Análises Clínicas, Departamento de Farmácia, Universidade Federal do Paraná, Curitiba, Brazil; 7 Câmara de Educação do Campo, Universidade Federal do Paraná (UFPR), Setor Litoral, Matinhos, Brazil; 8 Present Address: Departamento de Genética, Universidade Federal do Rio Grande do Sul (UFRGS), Porto Alegre, Brazil

**Keywords:** Development, Cardiovascular diseases, Risk factors

## Abstract

**Background:**

This study investigated butyrylcholinesterase (BChE) activity and its genetic variants (−116, A, and K) in Afro-Brazilian individuals living in two African-derived communities (quilombos) in southern Brazil. Parameters, including obesity, glucose, and lipid profile, were compared with those of Euro-Brazilian individuals from the same region. The study aimed to characterize population parameters and assess their influence on factors involved in metabolic syndrome, obesity, and type 2 diabetes mellitus (TDM2).

**Methods:**

A total of 198 Afro-Brazilians and 114 Euro-Brazilians participated. BChE activity was measured using the Ellman method, and variants were genotyped by PCR-SSP. Statistical analyses included *t*-test or Mann–Whitney, ANOVA, chi-square or Fisher’s exact test, correlations, and age- and sex-adjusted regression models.

**Results:**

The mean BChE activity in the Quilombola population was 3.71 U/mL and was significantly higher in people with TDM2 and/or obesity (*p* = 0.016), and associated with triglycerides, total cholesterol, overweight, and male sex. A significant interaction between triglycerides and glycated hemoglobin was observed (*p* = 0.016). BChE activity was higher in Quilombolas than in Euro-Brazilians, regardless of metabolic status, suggesting ethnic influence. BCHE gene variants (A, −116, and K) showed frequencies similar to other populations. Although enzymatic activity varied among haplotypes, differences were not statistically significant. These results highlight the relevance of clinical and ethnic factors in interpreting BChE activity.

**Conclusions:**

This study revealed higher BChE activity and TDM2 prevalence in Afro-Brazilian populations compared to Euro-Brazilians, and these differences correlate with non-genetic factors. The BCHE genetic variants investigated showed no robust associations after correction for multiple testing, suggesting that their effects on metabolic traits may be subtle and context-dependent. This is the first study to quantify BChE activity in Afro-Brazilian Quilombola populations and the second in Black individuals in Brazil, highlighting the importance of considering genetic and ethnic factors in metabolic risk assessment.

## Introduction

Butyrylcholinesterase (BChE), an enzyme primarily synthesized by the liver and widely distributed across tissues, plays an important role in the cholinergic anti-inflammatory pathway by hydrolyzing acetylcholine and modulating immune responses [1]. Alterations in BChE activity were observed in inflammatory conditions such as diabetes mellitus and cardiovascular and neurological diseases [2]. BChE activity is a biomarker for monitoring inflammation, surgical infections, and anastomotic leaks [3].

BChE has been proposed as a secondary marker in metabolic syndrome (MS) due to its complex biochemical interactions influencing obesity, insulin resistance, and dyslipidemia [4, 5]. BChE hydrolyzes ghrelin, converting it into an inactive form, which impacts satiety and energy intake [6]. However, studies suggest that the relationship between BChE and body weight may be complex, with a positive correlation dependent on the context [7]. Additionally, BChE is implicated in the pathogenesis of TDM2, contributing to insulin resistance through amyloid fibrils that affect pancreatic β-cells [8]. These associations highlight the potential of BChE as a biomarker for obesity and related metabolic diseases [9]. BChE is encoded by the *BCHE* gene, located on chromosome 3 (3q26.1-3q26.2), approximately 64 kb long, and has four exons (Supplementary Fig. 1) [10]. Mutations in the gene can lead to butyrylcholinesterase congenital deficiency (BCHED) (OMIM #617936), a rare, autosomal recessive condition characterized by prolonged sensitivity to muscle relaxants such as succinylcholine and mivacurium. While often asymptomatic, individuals with BCHED, particularly those with more than 70% enzyme reduction, can experience prolonged apnea and severe neuromuscular blockade, including the risk of death, due to the slow hydrolysis of these drugs [11–14]. The Food and Drug Administration (FDA) recommends caution with succinylcholine use in individuals with reduced BChE activity, especially in homozygotes for the atypical (A) variant [15]. Despite the pharmacogenetic importance of these variants, testing for BChE deficiency is carried out by biochemical methods only offered to patients who have a personal or family history of prolonged post-succinylcholine neuromuscular blockade [16, 17].

The combined presence of the −116 and K variants has been reported to reduce BChE activity, suggesting a functional interaction between these polymorphisms [1, 18]. The frequencies of these variants vary across populations and geographic regions, and except for the K variant, they are rare. Together, these genetic variations contribute to differences in BChE activity and may modulate susceptibility to metabolic and pharmacological outcomes [16, 19–21].

In this context, we aimed to genotype and analyze three functional *BCHE* variants—A (rs1799807), *K* (rs1803274), and −116 (rs1126680)—and to determine their frequencies, along with BChE enzymatic activity, in Afro-Brazilians living in Quilombola communities in Lapa, Paraná, southern Brazil. Quilombos are communities established by formerly enslaved Africans and their descendants, predominantly in rural and isolated areas. These communities represent enduring symbols of resistance to slavery and currently face socioeconomic vulnerabilities that can limit access to health care and elevate the prevalence of chronic diseases. Simultaneously, quilombos are centers of Afro-Brazilian culture preservation, fostering community identity [21–23]. Furthermore, we investigate the potential associations between these genetic variants and BChE activity with components of MS and the high prevalence of TDM2 observed in these communities. This research is one of the few investigations quantifying BChE activity in a Black population and, to our knowledge, the first in a Quilombola community, thereby addressing a gap in the representation of these populations in genetic and metabolic research.

## Methods

### Research participants

We appreciate the participation of the 198 adults (≥18 years) from the Quilombola communities of Feixo and Restinga, located in the city of Lapa, Paraná State, southern Brazil. The mean age of participants was 43.0 ± 15.2 years (mean ± SD).

All participants provided written informed consent prior to enrollment. The study received ethical approval from the Research Ethics Committee of the Federal University of Paraná (CEP/SD 4.639.630/CAAE 42761521.9.0000.0102). Self-reported skin color was recorded according to the categories of the Brazilian Institute of Geography and Statistics (IBGE): White (Branco), Admixed (Pardo), and Black (Preto) [24]. Consistent with IBGE classification, individuals identifying as Admixed or Black were considered Afro-descendants. Structured questionnaires collected sociodemographic (sex, age, birthplace) and medical data. BMI was calculated from measured height and weight. Eligibility required informed consent, blood samples, bioimpedance, and completed questionnaires.

As a comparison for BChE quantification, 114 frozen plasma samples from Euro-Brazilians (self-declared white, non-Quilombola individuals, living in the same geographical region—Paraná state) were utilized. Data on age, sex, glycemia, and lipid profile for this comparison group had been previously collected [25].

### Hematological and biochemical tests

Serum glucose levels were measured using the enzymatic hexokinase method with a Labmax 400 automated analyzer. The lipid profile was obtained using the enzymatic trinder method on the same analyzer. Glycated hemoglobin (HbA1c) in whole blood was measured using immunoturbidimetry, also in the same equipment. Glucose tolerance tests, when performed, utilized plasma glucose measurements based on the same enzymatic hexokinase principle. All biochemical analyses were conducted within 24–72 h of blood collection, during which time samples were stored at 4 °C.

### BChE activity

The enzymatic activity of butyrylcholinesterase (BChE) was determined by absorbance using a microplate spectrophotometer (TECAN® Infinite 200), following the technique described by Dietz et al. [26], modified by Evans and Wroe [27]. This assay measures the hydrolysis of propionylthiocholine by BChE, yielding propionic acid and thiocholine. The produced thiocholine then reacts with 5,5’-dithio-bis (2-nitrobenzoic acid) (DTNB) to form 5-thio-2-nitrobenzoate, a yellow chromophore with maximum absorbance at 410 nm. The assay was performed at 25 °C. Plasma samples, collected in ethylenediaminetetraacetic acid (EDTA) tubes and stored at −80 °C, were thawed and initially diluted 1:25 (10 μL plasma in 240 μL ultrapure water). Each sample was analyzed in triplicate in 96-well microplates. Each well received 220 μL of a phosphate buffer containing DTNB, followed by 25 μL of the diluted plasma sample. Control wells omitted the plasma sample. The reaction was initiated by the addition of 5 μL of the propionylthiocholine substrate immediately before the kinetic readings. Absorbance was measured at 410 nm at four time points: an initial reading (*A*0) and subsequent readings at 1-min (*A*1), 2-min (*A*2), and 3-min (*A*3) intervals. The rate of absorbance change per minute (Δ*A*/min) was calculated for each well using the formula:

$$\frac{{\mathrm{A}}/{\mathrm{min}}=({\mathrm{A}}1-{\mathrm{A}}0)+({\mathrm{A}}2-{\mathrm{A}}1)+({\mathrm{A}}3-{\mathrm{A}}2)}{3}$$

For each plasma sample, the mean Δ*A*/min value from the triplicates was used to determine BChE activity in *U*/*L*, and this value was multiplied by 34 (activity *U*/*L* = Δ*A*/min × 34). This factor is used to convert the change in absorbance, measured by the spectrophotometer, directly into enzymatic activity using the following calculation, adapted from Ellman et al. [28]:

$$\frac{{\mathrm{F}}={\mathrm{x}}.{\mathrm{y}}.\Delta {\mathrm{A}}/{\mathrm{min}}}{13.6.{\mathrm{z}}}$$

Where *x* is the cuvette volume (0.462 mL), *y* is the sample dilution factor [25], *z* is the sample volume (0.025 µL), and 13.6 is the molar absorptivity of DTNB in a 1 cm cuvette at 410 nanometers.

A control sample with known enzymatic activity was included in each assay run to ensure accuracy and reproducibility.

### Genotyping of *BCHE* polymorphisms

Genotyping for the *BCHE* A (rs1799807), K (rs1803274), and −116 (rs1126680) polymorphisms was performed using the Polymerase Chain Reaction with Sequence-Specific Primers (PCR-SSP)—also known as ARMS-PCR—which uses external primers for amplification and internal primers for allele specificity, as previously described [29]. For each polymorphism, four primers were designed (two outer and two inner), which were combined into three separate PCR reactions.

Mix 1 contained the forward outer and reverse outer primers and served as a positive amplification control, generating a larger amplicon. Mixes 2 and 3 were allele-specific reactions: Mix 2 combined the internal forward (allele-specific) primer with the external reverse primer, whereas Mix 3 combined the external forward primer with the internal reverse (allele-specific) primer (Supplementary Table 1). Reaction composition and cycling conditions are shown in Supplementary Tables 2 and 3.

PCR products were separated on a 2% agarose gel and subjected to electrophoresis at 100 V for 40 min for visualization of the resulting DNA bands under ultraviolet (UV) light. Genotypes were determined based on the presence or absence of allele-specific amplicons in Mix 2 and Mix 3, relative to the control amplicon in Mix 1 (Supplementary Fig. 2).

### Methods for classifying risk factors

Participants were classified as having TDM2 or pre-DM according to the guidelines of the Brazilian Diabetes Society (SBD) (Supplementary Table 4), taking into account self-reported prior physician diagnosis of TDM2, current use of hypoglycemic agents, glycated hemoglobin (HbA1c) levels, fasting plasma glucose levels, and, in some cases, a 2-h oral glucose tolerance test following the ingestion of 75 mg of glucose. Individuals were classified as pre-DM if they presented at least one altered test result. To ensure accurate classification of TDM2, particularly in the absence of clear clinical symptoms and without repeated confirmatory tests, a cautious approach was adopted, acknowledging that some individuals with altered results might be in the early stages of the disease. A history of physician-diagnosed hypertension and current use of antihypertensive medication were used to classify participants as having arterial hypertension (AH).

The classification of individuals with obesity and metabolic syndrome according to the guidelines of the Brazilian Association for the Study of Obesity and Metabolic Syndrome (ABESO). Individuals were classified as having obesity based on body mass index (BMI), together with measures of body fat distribution (Supplementary Table 5), which enhances the accuracy of identification. Metabolic syndrome was identified based on the presence of central obesity, defined by increased abdominal circumference, along with at least two of the following criteria: elevated triglyceride levels, reduced high-density lipoprotein cholesterol (HDL-cholesterol) levels, elevated systolic blood pressure (SBP) or diastolic blood pressure (DBP), and elevated fasting plasma glucose levels (Supplementary Table 6).

### Statistical analysis

Statistical analyses were performed in R (v. 4.3.1) via RStudio, using packages ggplot2 (graphics), dplyr (data handling), and genetics (genetic analysis). All tests were two-sided with significance at *p* < 0.05. Normality was assessed by Shapiro–Wilk test. Two-group comparisons used Student’s *t* test or Mann–Whitney *U* test, while ANOVA compared >2 groups. Categorical variables were analyzed via Chi-square or Fisher’s exact test, and continuous variables correlated using Pearson’s or Spearman’s coefficients. SNP-level and haplotype-based analyses evaluated associations between BCHE variants, linkage disequilibrium (LD), and metabolic traits. Continuous outcomes were assessed via age- and sex-adjusted linear regression under additive and dominant models. Diabetes status was analyzed using age- and sex-adjusted logistic regression. Multiple testing was corrected using the False Discovery Rate (FDR).

## Results

### Population characteristics

The Afro-Brazilian cohort is predominantly female (*n* = 140, 71%) and self-identified as Admixed (*n* = 116, 59%) or Black (*n* = 62, 31%) (Table 1). The prevalence of TDM2 was 20.7%, approximately double the national prevalence of 10.2% reported by the Surveillance of Risk and Protective Factors for Chronic Diseases by Telephone Survey (VIGITEL BRASIL, 2023). Similarly, the prevalence of AH was 32.8%, exceeding the national rate of 27.9%. A high proportion of participants with TDM2 (64.28%) and pre-DM (66.66%) presented AH, a comorbidity known to elevate the risk of cardiovascular complications, complicate treatment regimens, increase healthcare costs, and reduce life expectancy [30].

**Table 1:** Comparison of anthropometric and clinical parameters between the study groups.

Variable	No diabetic (*n* = 139)	SI units	Pre-diabetic (*n* = 18)	SI units	TDM2 (*n* = 41)	SI units	*p* value (Non-diabetics versus Pre-DM)	*p* value (TDM2 versus non-diabetics)
Age (years)	36		50		56		<0.001	<0.001
Sex (*n*)								
Female	96		11		33		0.679	0.219
Male	43		7		8			
Skin color (*n*)								
White	15		2		3		1	0.768
Black	40		6		16		0.796	0.376
Admixed	84		10		22		1	0.768
Glucose (mg/dL)	79	4.38	89	4.94	109	6.05	<0.001	<0.001
HbA1c (%)	5.2	33	5.78	40	6.95	52	<0.001	<0.001
Cholesterol (mg/dL)	190	4.94	210	5.43	190	4.94	0.058	0.987
Triglycerides (mg/dL)	95	1.07	135	1.52	126	1.42	0.007	<0.001
HDL (mg/dL)	50	1.29	49.5	1.28	55	1.42	0.137	0.064
LDL (mg/dL)	108	2.79	129	3.33	111	2.87	0.605	0.043
BChE (U/mL)	3.59		3.35		4.20		0.043	0.100
Waist circumference (cm)	92.5		103		105		0.005	<0.001
Belly circumference (cm)	97.9		108		109		0.007	<0.001
Hip circumference (cm)	105		109		109		0.149	0.022
Obesity (By BMI)								
Underweight	18.1		–		–		–	–
Normal or eutrophic	22.6		19.8		23.1		0.152	0.752
Overweight or pre-obesity	28.1		27.8		27.6		0.503	0.320
Obesity I	32.2		32.4		31.8		0.799	0.349
Obesity II	37.1		36.3		37.1		0.405	0.898
Severe obesity	41.4		–		–		–	–
Visceral fat	8		10		12		<0.001	<0.001
Waist-hip ratio	0.882		0.945		0.962		0.001	<0.001
Waist-height ratio	0.572		0.643		0.661		0.001	<0.001
BMI	27.9		30.7		32.1		0.041	<0.001
Muscle mass	25.3		25.9		27.1		0.316	0.037

Continuous variables are presented as median values; categorical variables as *n* (%). *p* values were calculated using Mann–Whitney or chi-square tests. SI units are shown for glucose, lipids (mmol/L), and HbA1c (mmol/mol).

*TDM2* type 2 diabetes mellitus, *HDL* high-density lipoprotein, *LDL* low-density lipoprotein, *BMI* body mass index, *HbA1c* glycated hemoglobin.

Of the participants, 78.7% were individuals with overweight or obesity, higher than the 60.8% reported by the National Health Survey (PNS) of IBGE. Among these, 43.8% were classified as at high risk for developing obesity-related comorbidities, including TDM2, HA, and dyslipidemia. However, the prevalence of metabolic syndrome was 10.8% in the study population, lower than the 33% reported in a meta-analysis of Brazilian studies [31].

### BChE activity in the Quilombo communities

**Fig. 1:**
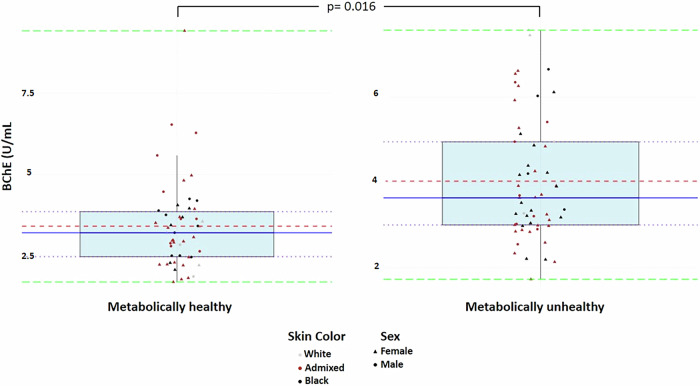
Enzymatic activity of BChE in metabolically healthy and metabolically unhealthy Quilombolas.

The average BChE activity was 3.71 U/mL (range: 1.15–9.42 U/mL). Notably, it was significantly higher in individuals with TDM2 and/or obesity (4.02 U/mL), compared to the individuals without TDM2 or obesity (3.41 U/mL; *p* = 0.016) (Fig. 1). These results underscore the need to consider clinical factors such as the presence of obesity and diabetes in addition to sex and age when establishing reference intervals for BChE activity in this population.

A significant correlation was observed between BChE activity and both triglycerides (TG) and total cholesterol levels (adjusted *R*² = 0.071, *p* = 0.002). Specifically, BChE activity showed a positive association with TG (*p* = 0.011) and a negative correlation with total cholesterol (*p* = 0.005). When the sample was categorized by obesity status, individuals living with overweight or obesity had a significantly higher mean BChE activity (3.62 U/mL) compared to the non-overweight group (3.21 U/mL; *p* = 0.03). Participants with TDM2 also exhibited significantly higher mean BChE activity (4.20 U/mL) compared to those without TDM2 (3.59 U/mL; *p* = 0.043).

Moreover, men had significantly higher BChE levels than women (4.26 vs. 3.53 U/mL, *p* = 0.001), and there were no significant differences between self-reported color groups (*p* = 0.703).

A linear regression model incorporating interaction terms revealed a statistically significant interaction between triglyceride (TG) and glycated hemoglobin (HbA1c) on BChE activity (*p* = 0.016), suggesting a potential combined influence of these variables. The interaction between Body Mass Index (BMI) and sex was not statistically significant.

In summary, BChE activity in this population is associated with lipid profile alterations (specifically TG and total cholesterol), TDM2, excess weight, and sex. The interaction between triglycerides and HbA1c suggests a shared pathophysiological pathway affecting BChE levels. These results support BChE as a complementary biomarker of metabolic disturbances related to metabolic syndrome.

### BChE activity is higher in the Quilombo communities than in Euro-Brazilians

BChE activity in the Quilombola populations was compared to a sample of 114 Euro-Brazilians (self-declared white non-Quilombola individuals) (Table 2). While the Euro-Brazilian plasma samples had a longer storage duration at −80 °C compared to the Quilombola samples, both were quantified for the BChE enzyme using the same methodology. Supporting the reliability of the comparison, previous studies have demonstrated the stability of biomarkers in plasma stored at −80 °C for up to 13 years, and existing literature suggests BChE enzyme activity remains stable under such conditions with minimal freeze-thaw cycles [32, 33].

**Table 2:** Comparison of anthropometric and clinical parameters between Quilombolas and Euro-Brazilians.

Variable	Quilombos (*n* = 198) Median (min–max)	SI units (mmol/L) Median (min–max)	Euro-Brazilians (*n* = 114) Median (min–max)	SI units (mmol/L) Median (min–max)	*p* value
Age (years)	42 (18–88)		46 (26–68)		0.697
Sex					0.915
Male	58		40		0.711
Female	140		74		0.474
Glucose (mg/dL)	83 (50–426)	4.6 (2.8–23.6)	82.5 (62–257)	4.58 (3.44–14.26)	0.457
Cholesterol (mg/dL)	207 (46–426)	5.35 (1.19–11.1)	190 (69–331)	4.9 (1.78–8.56)	0.004
Triglycerides (mg/dL)	106 (30–539)	1.19 (0.34–6.1)	111 (32–427)	1.25 (0.36–4.82)	0.389
HDL (mg/dL)	54 (17–113)	1.4 (0.44–2.92)	50 (30–102)	1.29 (0.78–2.64)	0.026
LDL (mg/dL)	112 (27.4–223)	2.9 (0.71–5.77)	128 (65–257)	3.31 (1.68–6.65)	<0.001
Waist circumference (cm)	95.5 (58–129)		83 (63.5–129)		<0.001
Hip circumference (cm)	106 (73–139)		101 (84.5–128)		0.010
BMI	29 (15.3–42)		26.1 (17.9–44)		<0.001
BChE (U/mL)	3.45 (1.15–9.42)		3 (1.12–6.75)		<0.001
Low	2.23 (1.15–2.71)		2.12 (1.12–2.53)		0.027
Medium	3.45 (2.80–4.36)		3.02 (2.56–3.53)		<0.001
High	5.34 (4.38–9.42)		3.95 (3.55–6.75)		<0.001

Data are presented as median (min–max). Laboratory measurements were originally obtained in mg/dL for glucose and lipid variables. An additional column presents the corresponding values converted to SI units (mmol/L for glucose and lipids). The classification of BChE activity as “Low”, “Medium”, or “High” was based on group-specific quartiles. *p* values refer to comparisons between groups.

*HDL* high-density lipoprotein, *LDL* low-density lipoprotein, *BMI* body mass index.

Consistent with observations in the Quilombola communities, within the Euro-Brazilian group, men exhibited higher mean BChE activity and body weight compared to women. Furthermore, BChE activity was significantly higher in Euro-Brazilians with obesity compared to their counterparts without obesity (3.21 vs. 2.72 U/mL, *p* = 0.002). These findings corroborate the influence of sex and body weight on BChE enzymatic activity. Further analysis in the Euro-Brazilian group revealed significant positive correlations with body mass index (BMI) (*r* = 0.386, *p* = 0.00002) and waist circumference (*r* = 0.315, *p* = 0.024). However, no significant correlations were observed with cholesterol, LDL, or triglycerides in this group.

Among the Euro-Brazilian subjects, a similar pattern to the Quilombola group was found: BChE activity was significantly higher in people living with overweight or obesity and classified as metabolically unhealthy (3.29 U/mL), compared to metabolically healthy people, who did not present either condition. (2.73 U/mL; *p* = 0.001) (Supplementary Table 7). Direct comparisons between the metabolic healthy subgroups of both populations showed significantly higher mean BChE activity in Quilombola participants (3.21 U/mL) compared to metabolic healthy Euro-Brazilians (2.73 U/mL; *p* = 0.003). Similarly, among the metabolic unhealthy subgroups, Quilombola participants (3.64 U/mL) exhibited significantly higher mean BChE activity compared to unhealthy Euro-Brazilians (3.29 U/mL; *p* = 0.019). These findings indicate that the Quilombola population tends to have higher BChE enzyme levels regardless of their metabolic health status. These results underscore the importance of considering factors such as ethnicity, clinical condition, sex, and age when interpreting enzymatic activity and establishing population-specific reference values.

### Frequencies of *BCHE* genetic variants in Quilombola communities and worldwide populations

**Fig. 2:**
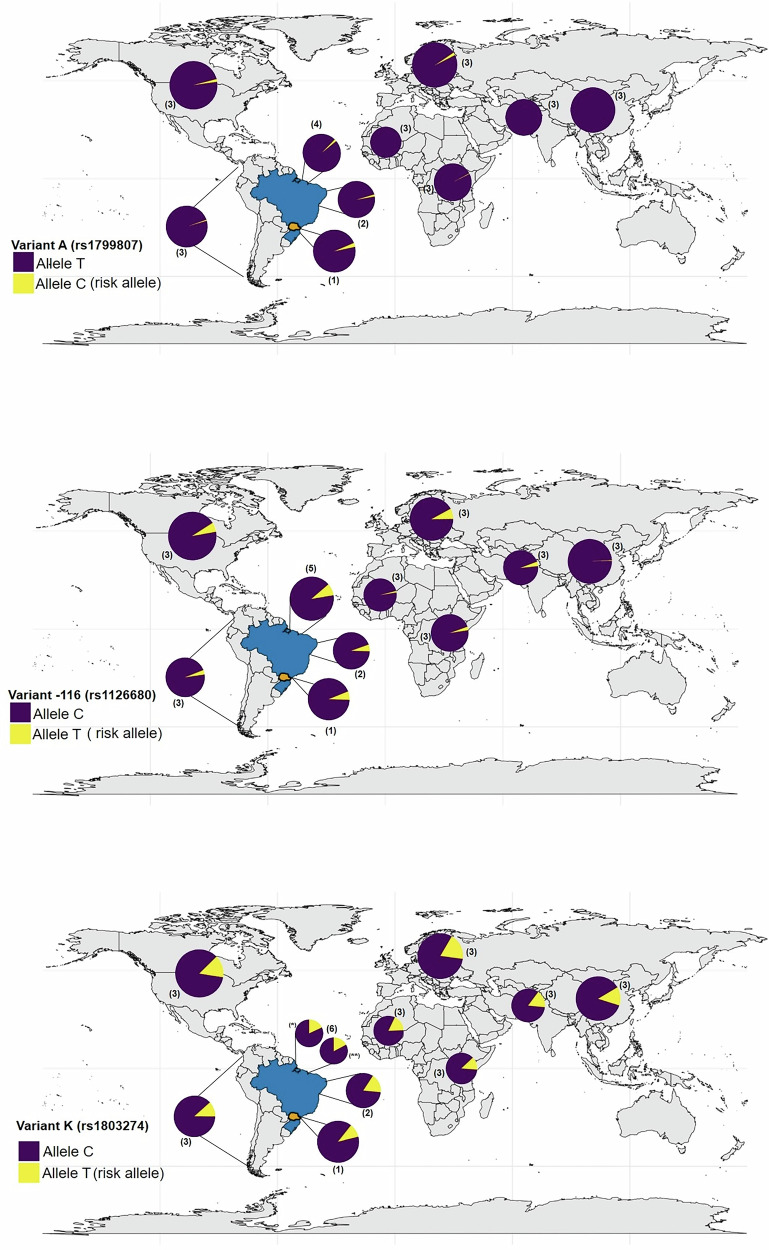
Distribution of the A, K and −116 polymorphisms in different populations. (1) Quilombola communities studied; (2) Online Archive of Brazilian Mutations (AbraOM); (3) 1000 Genomes Project; (4) Mikami, Liya R. ab; 2008; (5) Lupe Furtado-Alle, 2008. (6) Souza, R. L. R., 1998 - * Euro-Brazilians, **Afro-Brazilians.

Genotyping was successfully performed on 163 Quilombola participants for the *−116* (rs1126680) on 164 participants for the A (rs1799807) polymorphism, and on 165 participants for the K (rs1803274) polymorphism (Supplementary Table 10). All three polymorphisms were found to be in Hardy-Weinberg equilibrium within the analyzed sample. Significant linkage disequilibrium (LD) was observed between the K (rs1803274) and −116 (rs1126680) polymorphisms (*D*′ = 0.586, *R*² = 0.481, *p* < 0.001). The frequencies found in the Afro-Brazilian population did not differ significantly from those in other populations studied (Fig. 2).

**Fig. 3:**
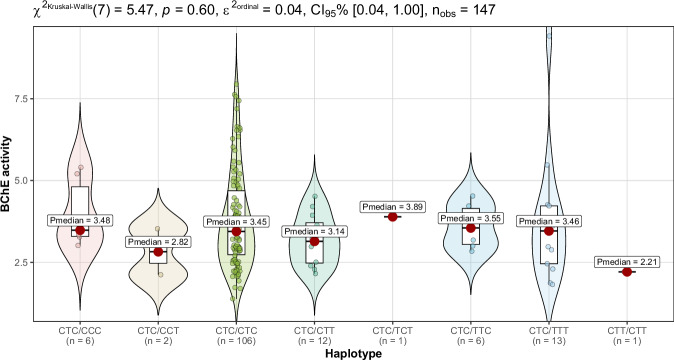
Variation in BCHE activity between haplotypes. Haplotype frequencies were inferred using the expectation–maximization algorithm. Haplotypes are displayed as diplotypes, and BChE activity was available for 147 individuals. Comparisons between haplotype groups were performed using the Kruskal–Wallis test, and no statistically significant differences were observed (*p* = 0.60). Haplotypes represented by one or two individuals should be interpreted with caution.

Haplotype analysis of BChE variants was also conducted in 163 individuals (Supplementary Table 10). BChE activity data were available for 147 individuals. The most frequent haplotype was the wild-type combination, lacking any of the identified risk alleles (−116*C, A*T, K*C) (Fig. 3). Homozygosity for risk alleles was only observed for the K variant (*n* = 1). However, several individuals carried multiple risk alleles: −116 and K (*n* = 13), A and K (*n* = 2), and −116, A, and K (*n* = 1). In total, 41 individuals carried at least one risk allele. Median BChE activity across haplotypes ranged from 2.21 U/mL to 3.89 U/mL. The lowest median activity (2.21 U/mL) was observed for one individual with the CTT/CTT haplotype (homozygous for the K variant), while the highest median activity (3.89 U/mL) was found in the CTC/TCT haplotype (heterozygous for all three variants). Overall, no statistically significant differences in BChE activity were observed between the haplotype groups (*p* = 0.60). Although post hoc tests suggested some differences, these should be interpreted cautiously due to the small sample size of certain haplotype groups.

Formal SNP-level association analyses were performed using age- and sex-adjusted additive and dominant genetic models; no robust associations with continuous metabolic traits were observed after correction for multiple testing (Supplementary Table 8). In age- and sex-adjusted logistic regression analyses, none of the *BCHE* polymorphisms showed significant associations with diabetes mellitus status under either additive or dominant genetic models (Supplementary Table 9). As a sensitivity analysis, logistic regression models were additionally adjusted for BMI, yielding results consistent with the primary analyses. Haplotype-based analyses focused on BChE enzymatic activity, a proximal functional phenotype, and did not reveal significant differences between haplotype groups.

## Discussion

Our study revealed a significantly elevated prevalence of people with TDM2 (20.7%), people with obesity (78.68%) in the analyzed Quilombola populations compared to the Brazilian national averages (10.2% and 55.4%, respectively) [34]. The strong co-occurrence of TDM2 and HA (64.28% of TDM2 and 66.66% of pre-DM participants also had HA) is particularly concerning, as this synergistically increases the risk of cardiovascular complications and other chronic diseases [35]. The high prevalence of people with obesity, especially visceral adiposity, likely contributes to a state of chronic low-grade inflammation, disrupting glucose metabolism, impairing pancreatic β-cell function, and exacerbating insulin resistance and hyperglycemia [36, 37]. This clustering of TDM2, obesity, and metabolic syndrome in individuals not only elevates cardiovascular risk but also diminishes quality of life and places a substantial burden on the healthcare system [38, 39].

The age-dependent increase in TDM2 prevalence observed in the Quilombola population, absent among individuals aged 18 to 27 with a sharp rise after 48 years and exceeding 50% in those over 58, aligns with established literature indicating an increased diabetes incidence from the age of 45 onwards [40]. The notable frequency of pre-DM (9.1%), predominantly in women, and the earlier onset of pre-DM in men (average 48 years) warrant further investigation into potential sex-specific risk factors within this population.

A key finding of our study was the significantly higher BChE activity in the Quilombola participants compared to the Euro-Brazilians, even after accounting for similar metabolic profiles. This suggests that genetic factors linked to ancestry, coupled with unique environmental and social determinants prevalent in Quilombola communities (such as dietary patterns, psychosocial stress, and healthcare access), may influence BChE levels. The elevated BChE activity in Quilombolas does not appear solely driven by lipid profiles but also correlates with the higher prevalence of people with obesity and TDM2, potentially indicating an adaptive enzymatic response to metabolic dysregulation [14, 41]. Furthermore, the sustained higher BChE activity in metabolically healthy Quilombolas underscores the necessity for population-specific reference intervals for this enzyme, highlighting its potential as a metabolic marker sensitive to both genetic and environmental specificities.

Our results corroborate existing literature by demonstrating a positive correlation between BChE activity and triglycerides (TG) and a negative correlation with total cholesterol [42, 43]. The positive association with TG suggests BChE’s involvement in lipolytic processes, particularly in conditions of increased cardiovascular risk like metabolic syndrome and type 2 diabetes [3, 14, 44]. The inverse relationship with total cholesterol may reflect a more complex interplay between lipoproteins and BChE, potentially influencing not only lipolysis but also cholesterol transport and distribution, a mechanism requiring further elucidation.

Higher enzyme activity was also observed in men, which may be related to physiological and hormonal differences, such as testosterone levels, which directly affect lipid metabolism and visceral fat distribution [45, 46]. Furthermore, the significant interaction between TG and HbA1c suggests a synergistic effect of hyperglycemia and hypertriglyceridemia on lipid metabolism, impacting BChE activity. These findings support the potential of BChE as an indirect biomarker for assessing metabolic and cardiovascular risk, especially in populations with lipid and glycemic disturbances [19].

A previous study found no significant differences in activity between Black and White populations [47]. Variants of the gene, on the other hand, tend to be less frequent in Blacks [48, 49]. The conflicting data in the literature regarding BChE activity across different populations and also between sexes [48–52] underscores the importance of population-specific studies, particularly in underrepresented groups like Black populations, to disentangle the complex interplay of genetics, diet, and lifestyle in determining BChE levels. Our findings of higher BChE activity in Quilombolas contribute valuable data to this area.

Regarding *BCHE* polymorphisms, formal association analyses did not reveal robust associations with metabolic traits or diabetes mellitus after correction for multiple testing. Nevertheless, nominal trends were observed for specific variants in relation to blood glucose levels and body weight, in line with existing evidence implicating BChE in energy homeostasis [9, 20, 53]. These findings suggest that common *BCHE* variants are more likely to exert subtle, context-dependent effects rather than acting as major determinants of metabolic outcomes. The moderate association between the K variant and reduced enzyme activity observed in individuals with TDM2 further indicates that genetic effects may be modulated by age, metabolic status, lifestyle, and environmental factors. Differences in allele frequencies across self-reported color groups likely reflect population history and evolutionary processes, which may contribute indirectly to heterogeneity in metabolic susceptibility [54, 55].

Our findings underscore the importance of considering functional biomarkers, alongside genetic and population factors, including ancestry and environmental context, when assessing metabolic risk. Further research is warranted to better understand the intricate interactions between *BCHE* genetic variants, enzyme activity, and metabolic conditions within diverse populations, especially in the African diaspora.

Beyond its peripheral metabolic role, BChE has been increasingly recognized as a multifunctional enzyme at the interface between metabolic regulation, neuroendocrine signaling, and behavioral pathways. At the molecular level, evidence linking *BCHE* to neurochemical and metabolic mechanisms primarily derives from functional, experimental, and animal studies, whereas integrated molecular profiling and human mechanistic data remain limited. In contrast, large-scale gene expression or omics-based profiling studies focusing on BChE in neuro-metabolic contexts remain scarce [1]. Experimental and clinical evidence suggests that BChE may indirectly influence appetite regulation and energy balance through cholinergic mechanisms that modulate acetylcholine signaling, ghrelin metabolism, and reward-related pathways, including the endogenous opioid system [7, 56]. In addition, environmental and psychosocial factors such as chronic stress, lifestyle-related exposures, and seasonal influences on mood and eating behavior may modulate BChE activity and metabolic outcomes, particularly in socially vulnerable populations [57, 58]. Although the present study was not designed to assess neurobehavioral or seasonal effects directly, these pathways provide a biologically plausible framework linking BChE activity to the metabolic alterations observed in Quilombola communities and highlight important directions for future research.

## Conclusions

This study revealed an alarmingly high prevalence of people with diabetes and obesity in the Quilombola populations, significantly exceeding the national average, highlighting a critical need for targeted public health interventions in these communities. The observed associations between BChE activity and metabolic factors like triglycerides and cholesterol suggest the enzyme’s crucial role in relation to lipid and glucose metabolism, corroborating the potential role as a biomarker for metabolic risk, particularly in populations with dyslipidemia and glucose intolerance. Our investigation into *BCHE* gene variants highlights the importance of considering genetic background alongside ancestry and environmental context in metabolic risk assessment, while indicating that common variants may exert modest, context-dependent effects rather than acting as primary determinants of metabolic outcomes. The significant differences in BChE activity between Quilombolas and Euro-Brazilians emphasize the intricate interplay of genetics and environmental factors in shaping metabolic health. As the second study to quantify BChE activity in a Black population in Brazil and the first to do so in Quilombola communities, this research underscores the underrepresentation of these groups in scientific literature. These findings strongly advocate for the development and implementation of public health strategies specifically tailored to address the pronounced metabolic disparities in underrepresented populations, with a focus on the prevention and management of TDM2 and metabolic syndrome in Quilombola communities and other populations of the African diaspora.

## Supplementary information

Supplementary Material

## Data Availability

All data generated or analyzed during this study are available from the corresponding author on reasonable request. Access will be granted for non-commercial research purposes, respecting participant confidentiality.
