# Supplementary material for: Butyrylcholinesterase as a potential biomarker of metabolic imbalances in Afro-descendant populations
Source: Int J Obes (Lond). 2026 May 13;50(8):1751–60. doi: 10.1038/s41366-026-02095-2 (PMC13461358; doi:10.1038/s41366-026-02095-2)
Supplement: Supplementary file 1 [file 41366_2026_2095_MOESM1_ESM.docx]

**Figures**

**Supplementary Figure 1:** Structure of the *BCHE* gene and location of functional polymorphisms analyzed in this study

Schematic representation of the *BCHE* gene located on chromosome 3 (3q26.1–q26.2). The gene spans cytogenetic bands 3q26.1 to 3q26.2 and consists of four exons. The figure illustrates the relative positions of these variants along the *BCHE* gene structure and summarizes their reported functional effects.

**Supplementary Figure 2:** PCR-SSP genotyping strategy for *BCHE* polymorphisms.

For each *BCHE* polymorphism, genotyping was performed using three primers in separate PCR reactions (Mix 1–3). Mix 1 included external primers and served as a positive amplification control, whereas Mixes 2 and 3 combined external and internal allele-specific primers to enable selective allele detection. The schematic representation and representative gel shown correspond to the *BCHE* A variant (rs1799807). The same PCR-SSP strategy was used to genotype the K (rs1803274) and −116 (rs1126680) polymorphisms. The electrophoresis image is an actual agarose gel obtained during the experimental procedures of this study.

**Tables**

| **Variant** | **Mix** | **Primers** | **Sequence** | **Bases (pb)** |
| --- | --- | --- | --- | --- |
| - 116 (rs1126680) | 1 (Control) | Forward outer | 5' - GTCCCATTTGCAAGCTTCAGTAACTGTT - 3' | 313 |
|  |  | Reverse outer | 5' - AACTCTCGCGAGCTTTGTCAGTAACAGT - 3' |  |
|  | 2 (T) | Forward inner | 5' - TTACCCGATTCTCTGCAACAAAGATTGT - 3' | 173 |
|  |  | Reverse outer | 5' - AACTCTCGCGAGCTTTGTCAGTAACAGT - 3' |  |
|  | 3 (C) | Forward outer | 5' - GTCCCATTTGCAAGCTTCAGTAACTGTT - 3' | 196 |
|  |  | Reverse inner | 5' - ACATGATTTTCACTCCTTGCAAACTGTG - 3' |  |
| K (rs1803274) | 1 (Control) | Forward outer | 5' - GACCCACACAACTTTCTTTCTTGCTAGTG - 3' | 282 |
|  |  | Reverse outer | 5' - CTTTTCAGGCAAAGCGAGCTAATAACAA - 3' |  |
|  | 2 (T) | Forward inner | 5' - AATCCTGCTTTCCACTCCCATTCGGT - 3' | 203 |
|  |  | Reverse outer | 5' - CTTTTCAGGCAAAGCGAGCTAATAACAA - 3' |  |
|  | 3 (C) | Forward outer | 5' - GACCCACACAACTTTCTTTCTTGCTAGTG - 3' | 135 |
|  |  | Reverse inner | 5' - TTCCATATTTTACAGGAAATATTGATGCAG - 3' |  |
| A (rs1799807) | 1 (Control) | Forward outer | 5' - AGTGGCATTTTTTGGTTTAGGTGCTGGA - 3' | 281 |
|  |  | Reverse outer | 5' - GGATGAACTTGACAGTTTTTGGTGGCAC - 3' |  |
|  | 2 (C) | Forward inner | 5' - CTCTGATCCATGGAAGCCTGGAAAACTTTTAC - 3' | 198 |
|  |  | Reverse outer | 5' - GGATGAACTTGACAGTTTTTGGTGGCAC - 3' |  |
|  | 3 (T) | Forward outer | 5' - AGTGGCATTTTTTGGTTTAGGTGCTGGA - 3' | 147 |
|  |  | Reverse inner | 5' - AAATATGCAAATTCTTGCTGTCAGAACATCGA - 3' |  |

**Supplementary table 1:** sequences of primers used for genotyping *BCHE* polymorphisms and amplicon sizes

| **Variant** | **A (rs1799807)** | | **K (rs1803274)** | |
| --- | --- | --- | --- | --- |
| Reagents | Initial concentration | Final concentration | Initial concentration | Final concentration |
| Buffer | 1 | 1 | 1 | 1 |
| MgCl2 (mM) | 2.5 | 0.5 | 2.5 | 0.25 |
| dNTP (mM) | 0.2 | 1 | 0.2 | 1 |
| Primer forward (pmol/uL) | 0.2 | 0.2 | 0.2 | 0.2 |
| Primer reverse (pmol/uL) | 0.2 | 0.2 | 0.2 | 0.2 |
| Taq polymerase (U/uL) | 0.5 | 0.1 | 0.5 | 0.1 |
|  | **-116 (rs1126680) – (T)** | | **-116 (rs1126680) – (C)** | |
| Buffer | 1 | 1 | 1 | 1 |
| MgCl2 (mM) | 2.5 | 0.5 | 2.5 | 0.8 |
| dNTP (mM) | 0.2 | 1 | 0.2 | 1 |
| Primer forward (pmol/uL) | 0.2 | 0.2 | 0.2 | 0.2 |
| Primer reverse (pmol/uL) | 0.2 | 0.2 | 0.2 | 0.2 |
| Taq polymerase (U/uL) | 0.5 | 0.2 | 0.5 | 0.2 |

**Supplementary table 2:** Protocol for preparing the PCR-SSP reaction

| **Variant** | **A (rs1799807)** | | | **K (rs1803274)** | | |
| --- | --- | --- | --- | --- | --- | --- |
| Step | Cycling | Temperature (°C) | Time | Cycling | Temperature (°C) | Time |
| Initial denaturing | 1 | 94 | 3 min | 1 | 94 | 3 min |
| Denaturing | 27 | 94 | 45 s | 27 | 94 | 45 s |
| Annealing |  | 59 | 30 s |  | 54 | 30 s |
| Elongation |  | 72 | 45 s |  | 72 | 45 s |
| Final elongation | 1 | 72 | 5 min | 1 | 72 | 5 min |
|  |  |  |  |  |  |  |
|  | **-116 (rs1126680) – (T)** | | | **-116 (rs1126680) – (C)** | | |
| Initial denaturing | 1 | 94 | 3 min | 1 | 94 | 3 min |
| Denaturing | 27 | 94 | 45 s | 30 | 94 | 45 s |
| Annealing |  | 67.6 | 30 s |  | 63.2 | 30 s |
| Elongation |  | 72 | 45 s |  | 72 | 45 s |
| Final elongation | 1 | 72 | 5 min | 1 | 72 | 5 min |

**Supplementary table 3:** PCR-SSP cycling conditions

| **Parameters** | **Normal** | **Pre-DM** | **TDM2** |
| --- | --- | --- | --- |
| Fasting blood glucose (mg/dl) | < 100 | 100 to < 126 | ≥ 126 |
| Random blood glucose (mg/dl) | - | - | ≥ 200 |
| Two-hour post-OGTT blood glucose (mg/dl) | < 140 | 140 to < 200 | ≥ 200 |
| HbA1c (%) | < 5,7 | 5,7 to < 6.5 | ≥ 6.5 |

**Supplementary table 4:** Criteria applied for the diagnosis of TDM2 and Pre-diabetes

Source: Brazilian Diabetes Society (SBD), 2022. TDM2: Diabetes mellitus type 2; OGTT: oral glucose tolerance test; HbA1c: glycated hemoglobin.

**Supplementary Table 5:** International classification of obesity according to body mass index (BMI) and risk of disease (World Health Organization), which divides adiposity into degrees or classes

| **BMI (kg/m2)** | **Classification** | **Obesity grade/class** | **Risk of disease** |
| --- | --- | --- | --- |
| <18 | Underweight | 0 | Normal or Increased |
| 18,5 – 24.9 | Normal or Eutrophic | 0 | Normal |
| 25 – 25.9 | Overweight or Pre-obese | 0 | Slightly Increased |
| 30 – 34.9 | Obesity | I | Increased |
| 35 – 39.9 | Obesity | II | Highly Increased |
| ≥ 40 | Severe Obesity | III | Extremely Increased |

BMI: body mass index. Source: Brazilian Association for the Study of Obesity and Metabolic Syndrome Brazilian obesity guidelines, 2016.

**Supplementary Table 6:** Diagnostic criteria for metabolic syndrome in men and women, including abdominal circumference cut-off points as a mandatory criterion.

| **Mandatory criteria** | **Plus at least 2 of the 4 criteria** |
| --- | --- |
| Visceral obesity (waist circumference): Waist circumference measurements according to ethnicity (cm) for men and women: | Triglycerides ≥ 150 mg/dL or treatment |
|  |  |
|  |  |
| Europeans: ≥ 94 cm (men); ≥ 80 cm (women); | HDL < 40 mg/dL (men); < 50 mg/dL (women) |
| South Africans, Western Mediterranean, and Middle Eastern populations: Same as Europeans. | SBP ≥ 130 mmHg or DBP ≥ 85 mmHg or treatment |
|  |  |
| South Asians and Chinese: ≥ 90 cm (men); ≥ 80 cm (women); | Fasting glucose ≥ 100 mg/dL or previous diagnosis of diabetes |
| Japanese: ≥ 90 cm (men); ≥ 85 cm (women); | If fasting glucose > 99 mg/dL, an oral glucose tolerance test is recommended, but not necessary for the diagnosis of metabolic syndrome |
|  |  |
| South Americans and Central Americans: Follow the reference values for South Asians. |  |
|  |  |

SBP: Systolic Blood Pressure, DBP: Diastolic Blood Pressure, HDL. Source: Brazilian Association for the Study of Obesity and Metabolic Syndrome Brazilian obesity guidelines, 2016.

| **Statistic** | **Quilombolas metabolically healthy (n= 50)** | **Euro-Brazilians metabolically healthy (n=51)** | **p-value** | **Quilombolas metabolically unhealthy (n= 56)** | **Euro-Brazilians metabolically unhealthy (n=62)** | **p-value** |
| --- | --- | --- | --- | --- | --- | --- |
| Mean ± DP | 3.41 ± 1.38 | 2.71 ± 0.806 | 0.002 | 4.02 ± 1.44 | 3.29 ± 0.849 | 0.025 |
| Median | 3.21 | 2.71 |  | 3.58 | 3.27 |  |
| Confidence interval (95%) | 3.03 - 3.80 | 2.50 - 2.93 |  | 3.64 - 4.39 | 3.07 - 3.50 |  |
| Variation coefficient (%) | 40.5 | 29.7 |  | 35.8 | 25.9 |  |
| Maximum | 9.42 | 6.75 |  | 7.56 | 5.58 |  |
| Minimum | 1.70 | 1.12 |  | 1.73 | 1.14 |  |
| 1st quartile | 2.48 | 2.25 |  | 3 | 2.82 |  |
| 3st quartile | 3.86 | 3.05 |  | 4.89 | 3.79 |  |

**Supplementary Table 7:** Statistical analysis of bche enzyme activity among Quilombola groups and Euro-Brazilians

The sample was subdivided into healthy participants without metabolic disorders, such as obesity and diabetes, and unhealthy individuals, who have obesity and/or diabetes.

**Supplementary Table 8:** Association analyses between *BCHE* polymorphisms and continuous metabolic traits under additive and dominant genetic models

| **SNP** | **Outcome** | **Major genotype** | **Minor allele** | **n** | **Additive beta** | **Additive SE** | **Additive p** | **Additive q (FDR)** | **Dominant beta** | **Dominant SE** | **Dominant p** | **Dominant q (FDR)** |
| --- | --- | --- | --- | --- | --- | --- | --- | --- | --- | --- | --- | --- |
| rs1126680 (-116) | Blood glucose | C/C | T | 130 | 8.1 | 11.796 | 0.4936 | 0.9276 | 8.1 | 11.796 | 0.4936 | 0.9276 |
| rs1799807 (A) | Blood glucose | T/T | C | 132 | -14.641 | 17.547 | 0.4056 | 0.9276 | -14.641 | 17.547 | 0.4056 | 0.9276 |
| rs1803274 (K) | Blood glucose | C/C | T | 132 | 0.116 | 9.643 | 0.9904 | 0.9904 | 1.022 | 10.167 | 0.9201 | 0.9858 |
| rs1126680 (-116) | Body fat | C/C | T | 141 | -0.755 | 2.052 | 0.7135 | 0.9276 | -0.755 | 2.052 | 0.7135 | 0.9276 |
| rs1799807 (A) | Body fat | T/T | C | 143 | 0.712 | 2.864 | 0.8039 | 0.9276 | 0.712 | 2.864 | 0.8039 | 0.9276 |
| rs1803274 (K) | Body fat | C/C | T | 143 | 0.431 | 1.626 | 0.7915 | 0.9276 | 0.585 | 1.711 | 0.733 | 0.9276 |
| rs1126680 (-116) | Visceral fat | C/C | T | 141 | -0.34 | 0.811 | 0.6754 | 0.9276 | -0.34 | 0.811 | 0.6754 | 0.9276 |
| rs1799807 (A) | Visceral fat | T/T | C | 143 | 0.329 | 1.129 | 0.7708 | 0.9276 | 0.329 | 1.129 | 0.7708 | 0.9276 |
| rs1803274 (K) | Visceral fat | C/C | T | 143 | 0.637 | 0.639 | 0.3205 | 0.9276 | 0.696 | 0.672 | 0.3024 | 0.9276 |
| rs1126680 (-116) | BMI | C/C | T | 146 | -0.023 | 1.334 | 0.986 | 0.9904 | -0.023 | 1.334 | 0.986 | 0.986 |
| rs1799807 (A) | BMI | T/T | C | 148 | 0.947 | 1.895 | 0.6178 | 0.9276 | 0.947 | 1.895 | 0.6178 | 0.9276 |
| rs1803274 (K) | BMI | C/C | T | 148 | 1.336 | 1.068 | 0.2129 | 0.9276 | 1.424 | 1.123 | 0.2071 | 0.9276 |
| rs1126680 (-116) | Body weight | C/C | T | 146 | 2.741 | 4.009 | 0.4952 | 0.9276 | 2.741 | 4.009 | 0.4952 | 0.9276 |
| rs1799807 (A) | Body weight | T/T | C | 148 | 6.161 | 5.674 | 0.2794 | 0.9276 | 6.161 | 5.674 | 0.2794 | 0.9276 |
| rs1803274 (K) | Body weight | C/C | T | 148 | 7.03 | 3.172 | 0.0282 | 0.4237 | 7.332 | 3.339 | 0.0297 | 0.4455 |

Linear regression models were used to assess associations between BCHE polymorphisms and continuous metabolic traits under additive and dominant genetic models. Analyses were adjusted for age and sex. β represents the regression coefficient and SE the standard error. Outcome variables were analyzed on their original measurement scales. P-values were corrected for multiple testing using the false discovery rate (FDR) method.

| **SNP** | **Model** | **n** | **OR** | **LCL 95%** | **UCL 95%** | **p** | **q (FDR)** |
| --- | --- | --- | --- | --- | --- | --- | --- |
| rs1126680 (-116) | Additive (per minor allele) | 147 | 1.526 | 0.48 | 4.859 | 0.474 | 0.5576 |
| rs1126680 (-116) | Dominant (carrier vs non-carrier) | 147 | 1.526 | 0.48 | 4.859 | 0.474 | 0.5576 |
| rs1799807 (A) | Additive (per minor allele) | 149 | 0.574 | 0.09 | 3.675 | 0.5576 | 0.5576 |
| rs1799807 (A) | Dominant (carrier vs non-carrier) | 149 | 0.574 | 0.09 | 3.675 | 0.5576 | 0.5576 |
| rs1803274 (K) | Additive (per minor allele) | 149 | 0.672 | 0.254 | 1.773 | 0.4215 | 0.5576 |
| rs1803274 (K) | Dominant (carrier vs non-carrier) | 149 | 0.698 | 0.252 | 1.93 | 0.4884 | 0.5576 |

**Supplementary Table 9:** association between bche polymorphisms and diabetes mellitus using age- and sex-adjusted logistic regression models

Logistic regression models were used to estimate odds ratios (OR) and 95% confidence intervals (CI) for diabetes mellitus under additive (per minor allele) and dominant (carrier vs non-carrier) genetic models. Analyses were adjusted for age and sex. P-values were corrected for multiple testing using the false discovery rate (FDR) method.

| **A: GENOTYPIC AND ALLELIC FREQUENCIES** | | | | | |
| --- | --- | --- | --- | --- | --- |
| **SNP** | **Genotype** | **N** | **Frequency (%)** | **Allele** | **Allele frequency** |
| rs1799807 (A) | T/T | 153 | 93.3 | T | 0.966 |
|  | T/C | 11 | 6.7 | C | 0.034 |
|  | C/C | 0 | 0.0 |  |  |
| rs1803274 (K) | C/C | 132 | 80.0 | C | 0.897 |
|  | C/T | 32 | 19.4 | T | 0.103 |
|  | T/T | 1 | 0.6 |  |  |
| rs1126680 (−116) | C/C | 140 | 85.9 | C | 0.930 |
|  | C/T | 23 | 14.1 | T | 0.070 |
|  | T/T | 0 | 0.0 |  |  |
| **B: HAPLOTYPE FREQUENCIES** | | | | | |
| **Haplotype** | **rs1799807** | **rs1803274** | **rs1126680** | **Frequency** | |
| H1 | T | C | C | 0.848 | |
| H2 | T | T | C | 0.049 | |
| H3 | T | T | T | 0.043 | |
| H4 | T | C | T | 0.026 | |
| H5 | C | C | C | 0.024 | |
| H6 | C | T | C | 0.008 | |
| H7 | C | T | T | 0.002 | |

**Supplementary Table 10**: Genotypic, allelic and haplotype frequencies of *BCHE* variants in the Quilombola population

Genotype and allele frequencies were calculated by direct counting. Haplotype frequencies were estimated using the expectation–maximization algorithm. Sample sizes vary due to missing genotype data. All polymorphisms were in Hardy–Weinberg equilibrium (exact test, p > 0.05).
